# Supplementary material for: Factors and consequences associated with intimate partner violence against women in low- and middle-income countries: A systematic review
Source: PLoS One. 2023 Nov 8;18(11):e0293295. doi: 10.1371/journal.pone.0293295 (PMC10631698; doi:10.1371/journal.pone.0293295)
Supplement: S1 Table — (DOCX) [file pone.0293295.s002.docx]

| **S1.Table.** Detailed Quality assessment of the selected studies | Authors | Study objectives stated | Study population defined | Eligible participation rate at least 50%? | Participant selection and inclusion/exclusion criteria uniformity | Sample size sufficient and/or described | Exposure measured prior to outcome | Sufficient time frame for association between exposure and outcome | Inclusion exposure level | Exposure measures valid and reliable | Multiple exposures measurements | Outcome measures valid and reliable | Outcome assessor(s) blinded | Loss to follow up post-baseline 20% or less | Confounders measured and adjusted statistically between exposure and outcome | Overall rating |
| --- | --- | --- | --- | --- | --- | --- | --- | --- | --- | --- | --- | --- | --- | --- | --- | --- |
| 1. | Barnett et al. (2021) | √ | √ | √ | √ | √ | √ | √ | √ | √ | √ | √ | √ | √ | √ | Good |
| 2. | Bhowmik and Biswas (2022) | √ | √ | NA | √ | NA | √ | NA | √ | √ | NA | √ | √ | NA | √ | Good |
| 3. | Bondade et al. (2018) | √ | √ | NA | √ | √ | √ | NA | √ | √ | NA | √ | √ | NA | √ | Good |
| 4. | Coll et al. (2020) | √ | √ | NA | √ | NA | √ | NA | √ | √ | NA | √ | √ | NA | √ | Good |
| 5. | Hajian et al. (2018) | √ | √ | NR | √ | × | × | NA | NA | √ | × | √ | × | NR | √ | Fair |
| 6. | Jina et al. (2012) | √ | √ | NA | √ | × | √ | NA | √ | √ | NA | √ | √ | NA | √ | Fair |
| 7. | Jiwatram-Negrón et al. (2018) | √ | √ | NA | √ | × | √ | NA | √ | √ | NA | √ | √ | NA | √ | Fair |
| 8. | Meekers et al. (2013) | √ | √ | NA | √ | NA | √ | NA | √ | √ | NA | √ | √ | NA | √ | Good |
| 9. | Miller et al. (2022) | √ | √ | NA | √ | NA | √ | NA | √ | √ | NA | √ | √ | √ | √ | Good |
| 10. | Mootz et al. (2021) | √ | √ | NA | √ | × | √ | NA | √ | √ | NA | √ | √ | NA | √ | Fair |
| 11. | Pahn and Yang (2021) | √ | × | NR | √ | × | × | NA | NA | √ | × | √ | × | NR | √ | Poor |
| 12. | Sanni et al. (2021) | √ | √ | NA | √ | NA | √ | NA | √ | √ | NA | √ | √ | NA | √ | Good |
| 13. | Shaikh et al. (2017) | √ | √ | √ | √ | × | √ | NA | √ | √ | NA | √ | √ | NA | √  (Continued) | Fair |

| 14. | Sharma et al. (2019) | √ | √ | √ | √ | √ | √ | NA | √ | √ | NA | √ | √ | NA | √ | Good |
| --- | --- | --- | --- | --- | --- | --- | --- | --- | --- | --- | --- | --- | --- | --- | --- | --- |
| 15. | Leight et al. (2020) | √ | √ | √ | √ | × | √ | NA | √ | √ | NA | √ | √ | NA | √ | Fair |
| 16. | Wagman et al. (2018) | √ | √ | √ | √ | NA | √ | × | √ | √ | NA | √ | √ | NA | √ | Good |
| 17. | Amegbor and Rosenberg (2019) | √ | √ | √ | √ | NA | √ | √ | √ | √ | NA | √ | √ | √ | √ | Good |
| 18. | John et al. (2022) | √ | √ | NR | √ | × | √ | √ | × | √ | × | √ | × | NR | √ | Fair |
| 19. | McClintock et al. (2021) | √ | √ | NA | √ | NA | √ | √ | √ | √ | NA | √ | √ | NA | √ | Good |
| 20. | Memiah et al. (2021) | × | √ | NR | × | × | × | NA | × | × | × | √ | × | NR | √ | Poor |
| 21. | Oluwole et al. (2020) | √ | √ | √ | √ | √ | √ | √ | √ | √ | NA | √ | √ | NA | √ | Good |
| 22. | Rogathi et al. (2017) | √ | √ | √ | √ | √ | √ | NA | √ | √ | × | √ | × | √ | √ | Good |
| 23. | Soleimani et al. (2017) | √ | √ | NA | NA | NA | √ | NA | √ | √ | × | √ | × | NA | NA | Fair |
| 24. | Ibrahim et al. (2015) | √ | √ | √ | √ | NA | NA | NA | √ | √ | × | √ | × | NA | NA | Fair |
| 25. | Ahinkorah et al. (2018) | √ | √ | √ | √ | NA | √ | NA | √ | √ | NA | √ | √ | NA | √ | Good |
| 26. | Koen et al. (2014) | √ | √ | NA | √ | NA | √ | NA | √ | √ | NA | √ | √ | NA | √ | Good |
| 27. | Sabri et al. (2014) | √ | √ | √ | √ | NA | √ | NA | √ | √ | NA | √ | √ | NA | √ | Good |
| 28. | Kouyoumdjian et al. (2013b) | √ | √ | √ | √ | NA | √ | √ | √ | √ | √ | √ | √ | NA | √ | Good |
| 29. | Diamond-Smith et al. (2019) | √ | √ | NA | √ | NA | √ | NA | √ | √ | NA | √ | √ | NA | √ | Good |
| 30. | Hayati et al. (2011) | √ | √ | √ | √ | NA | √ | NA | √ | √ | NA | √ | √ | NA | √ | Good |

*√ - Yes × - No NA – Not Applicable due to cross-sectional population-based survey NR – Not Reported*
