# Supplementary material for: Factors and consequences associated with intimate partner violence against women in low- and middle-income countries: A systematic review
Source: PLoS One. 2023 Nov 8;18(11):e0293295. doi: 10.1371/journal.pone.0293295 (PMC10631698; doi:10.1371/journal.pone.0293295)
Supplement: Attachment — Submitted filename: Response to Reviewers.docx [file pone.0293295.s003.docx]

June 22, 2023

Abraham Salinas-Miranda
Academic Editor,
PLOS ONE

RE: PLOS ONE – Decision on Manuscript ID PONE-D-23-08947

Dear Editor,

We appreciate the opportunity to revise our manuscript titled "Factors and Consequences associated with Intimate Partner Violence against Women in Low- and Middle-Income Countries: A Systematic Review" for publication in PLOS ONE.

We have carefully addressed the points raised by the academic editor and reviewers and have incorporated the necessary changes into the original manuscript through a comprehensive revision. In the revised version, we have highlighted the reviewers' comments in blue and our responses in black font for clarity.

Furthermore, we confirm that all authors have thoroughly reviewed and revised the manuscript. We have made some changes to the revised version in order to improve its clarity.

We sincerely thank you for considering our paper and appreciate the valuable suggestions and feedback provided by the academic editor and reviewers. Their comments and suggestions have significantly improved the manuscript quality. We hope that the revised version will meet the standards for publication in your esteemed journal.

Thank you for your kind consideration.

Kind regards,

Lakma Gunarathne

Swinburne University of Technology

Victoria, 3122, Australia.

**Co-authors:**

1. Jahar Bhowmik, Department of Health Science and Biostatistics, Swinburne University of Technology, Australia.

2. Pragalathan Apputhurai, Department of Health Science and Biostatistics, Swinburne University of Technology, Australia.

3. Maja Nedeljkovic, Department of Psychological Sciences, Swinburne University of Technology, Australia.

**Factors and Consequences associated with Intimate Partner Violence against Women in Low- and Middle-Income Countries: A Systematic Review**

(PONE-D-23-08947)

(Please Note: Page numbers and line numbers in this document are based on Revised Manuscript with Track Changes file)

**Reviewer # 1**

**Introduction**

**Reviewer’s comment**

1. Overall, this introduction could benefit from revision and editing to make it more concise. There is a fair bit of repetition with the phrasing and statistics around the prevalence and impact of IPV and this section would benefit from being more succinct.

**Authors’ response**

Thank you for your valuable feedback and suggestion.

Based on your suggestion, we have edited the introduction section and have removed the repetitions to make it concise in the revised version of the manuscript (see pages 3-5, lines 68-70, 74-79, 82-84, and 101-102).

**Reviewer’s comment**

2. On Line 103 you note that there are not current systematic reviews of “factors and consequences of IPV against married women in LMICs” and in Line 105-106 you note “By finding associated risk factors and consequences of IPV in LMICs, this systematic review enhances understanding of IPV…” I think it is very important to be crystal clear if you were only looking at studies of married women or all women who experienced IPV?

**Authors’ response**

Thank you for your attention and picking up this typo. Following your suggestion, we have removed the word 'married' from Line 114 (see page 5) to avoid any inconsistency. We have also added the phrase "against women" in Line 117 (see page 5) to make it clear that the focus of this study is on all women in LMICs experiencing IPV, not exclusively married women. We have revised the whole manuscript to avoid this confusion.

**Reviewer’s comment**

3. Can you address in the introduction why the systematic review is just focused on women and not men who might also experience IPV? This may come out in limitations but as the introduction provides an overview of IPV and its consequences it would be helpful to address the fact that male victims of IPV are often not included in our discussions of IPV.

**Authors’ response**

Thank you for your insightful comment and suggestion. We agree that our introduction should emphasize why this systematic review focused on women not men regarding IPV. We have incorporated your suggestions into the introduction section and provided a reference based on the WHO Global and Regional estimates of violence against women report as below (see page 3, lines 61-66):

"Therefore, women are at a higher risk of experiencing IPV than men [5]."

This acknowledges the existing evidence regarding the higher risk of IPV among women than men, which has been supported by many past studies.

**Quality Assessment**

**Reviewer’s comment**

4. It is not clear why the NIH Quality Assessment Tool was used to evaluate the included studies. Please explain why this tool was selected.

**Authors’ response**

Thanks for your feedback regarding the use of the NIH Quality Assessment Tool in evaluating the included studies.

The NIH Quality Assessment Tool was chosen for assessing the quality of the included studies in our systematic review for several reasons. The NIH Quality Assessment Tool is a widely recognized and extensively used tool in healthcare research to assess methodological quality and bias risk of different study designs, including observational studies and cross-sectional studies (Ma, L. L., Wang, Y. Y., Yang, Z. H., Huang, D., Weng, H., & Zeng, X. T. (2020). Methodological quality (risk of bias) assessment tools for primary and secondary medical studies: what are they and which is better? *Mil Med Res*, *7*(1), 7. <https://doi.org/10.1186/s40779-020-00238-8>)

As our systematic review focused on identifying factors and consequences of intimate partner violence (IPV) among women, it was crucial that the included studies were of high methodological quality and validity. Moreover, all the selected studies are cross-sectional and observational in nature. Utilizing the NIH Quality Assessment Tool, we aimed to evaluate the methodological strengths and weaknesses of the included studies in a standardized and comprehensive manner. This enhanced the reliability and credibility of our findings.

Further, the NIH Quality Assessment Tool consists of a variety of key domains and criteria relevant to evaluating the quality of healthcare research studies, such as study design, sample size, data collection methods, statistical analyses, and results reporting. Using this tool, we were able to evaluate each study's quality and potential bias in a systematic manner, allowing us to make informed decisions regarding their inclusion in our review.

It is worth noting that the selection of the NIH Quality Assessment Tool was based on careful consideration of available tools and guidelines for quality assessment. We reviewed various options, including other widely used tools such as the JBI Critical Appraisal tool, Crowe Critical Appraisal tool, Strobe checklist and the Cochrane Collaboration's Risk of Bias Tool. Due to its compatibility with the study designs and outcomes included in the systematic review, the NIH Quality Assessment Tool was considered most appropriate for our specific research questions.

**Reviewer’s comment**

5. Please clarify that all studies were ranked using the quality assessment tool but that rankings on the quality assessment tool did not affect whether the study was included in the systematic review. I think that is what the process was but it is not clear in the current section.

**Authors’ response**

We appreciate your valuable feedback. We apologize for any confusion caused by the lack of clarity in this section. Regarding your concern, we confirm that all included studies underwent a quality assessment using the NIH Quality Assessment Tool. This quality assessment was carried out to determine the methodological rigor and potential bias of the studies included in the review. In the revised section, we have included explicit criteria for rating the studies. Furthermore, in the Results section, we have provided a summary of the study quality assessment findings. Based on the assessment, twenty-one studies were rated as good, nine studies as fair, and two studies as poor. We acknowledge that the two studies conducted by Pahn and Yang [22] and Memiah, Ah Mu [23] received poor ratings. However, despite their poor ratings, we decided to include these studies in the review due to their adequate power and utilization of cross-sectional self-reported opinion surveys. Therefore, regardless of their rankings, all studies were considered for inclusion. The following revisions were made to this section of the manuscript to make it clearer how our method worked.

“The National Institutes of Health (NIH) Quality Assessment Tool [21] was used to assess the quality of the included studies. This tool evaluated the observational, cohort and cross-sectional studies according to the following criteria: research question, study population, sufficient timeframe to see an effect, frequency of measure, different levels of the exposure of interest, sample size justification, outcome measures, blinding of outcome assessors, follow-up rate, statistical analyses, exposure measures and assessment, and study setting. In the assessment process, three independent authors rated the studies as “poor”, “fair”, or “good” based on the criteria described in the NIH Quality Assessment Tool. The articles that met less than six criteria were classified as “poor”, those that met 6 to 10 criteria were classified as “fair”, and those meeting more than 10 criteria were classified as “good”. Consistency among the three reviewers’ ratings was ensured, and any discrepancies were resolved by an independent fourth reviewer. This quality assessment was designed primarily to evaluate the methodological rigor and potential biases of the included studies. The final assessments were confirmed by all the authors and the quality assessment results of each article are presented in S1 Table 1.”

(See pages 7-8, lines 172-190).

**Results**

**Reviewer’s comment**

6. Line 224-228 is sharing a very important finding but it is very hard to follow the sentence structure. Please revise and perhaps divide into two sentences to make more clear.

**Authors’ response**

Thank you for your feedback about the clarity of the sentence in lines 224-228. We have revised the sentence according to your suggestion to improve its readability as below (page 14, lines 251-258):

“Results obtained from the selected studies indicate that the years of formal education for the male partners were associated with experience of IPV for the female partners. Women whose husbands had less than 9 years of formal education were found to be at a higher risk of exposure to IPV [47] as compared to women whose husbands had more than 9 years of formal education. Research has also demonstrated that male partners without tertiary education are more likely to be perpetrators of IPV [38].”

**Reviewer’s comment**

7. 277-279. Please rephrase “at a greater risk of developing IPV” as IPV is not something that is developed but rather experienced.

**Authors’ response**

Thanks for your suggestion. We have revised the sentence as follows in the revised version (page 17, lines 326-328):

“Another study reported that Kenyan women exposed to violence in their girlhood were at a greater risk of experiencing IPV than those who were not [29].”

**Discussion**

**Reviewer’s comment**

8. Line 431-440: The analysis of how economic status and age may impact the prevalence of IPV in LMICs being linked to girl children not going to school and being married young seems a bit reductive. Are there other factors that emerged from the literature review related to risk factors and the age of victims that could also be explored such as decision-making power, employment, social standing etc.?

**Authors’ response**

Thank you for your feedback. We have incorporated your suggestions and included the few other factors as follows in the revised version (See page 24, lines 498-502):

“Younger women are also found to be impacted by social norms, cultural expectations, gender discrimination, and financial vulnerabilities leading to fewer employment opportunities, lower social status, and limited decision-making power [59-62]. As a result, there is an imbalance of power within relationships, which increases the likelihood of young women being exposed to IPV.”

**Reviewer’s comment**

9. Line 478-481: The link between the exposure to childhood trauma and negative outcomes as an adult is well understood and there is extensive literature on this. I would like to see this section more robustly developed to cite to this literature and discuss why the findings on this from the systematic review is important.

**Authors’ response**

Thank you for your feedback and suggestion. According to your suggestion, we had revised this section added the following text in the revised version of the manuscript (see page 26, lines 547-564).

“In line with previous research, the current review demonstrated that witnessing and experiencing abuse as a child and childhood trauma conditions may result in a higher chance of having violent relationships in adulthood [29, 67, 68]. Childhood exposure to violence can result in a wide range of negative outcomes, including trauma, mental health issues, impaired social, emotional development, and interpersonal difficulties [69, 70]. Consequently, individuals who have been exposed to violent behaviour as a child may find it difficult to establish trust, intimacy, or resolve conflict in their adult relationships, which may increase the likelihood of experiencing or perpetrating IPV [71, 72]. In addition, the intergenerational transmission of violence theory shows that individuals who have been exposed to violence during their early childhood are more likely to continue their violent behaviour in response to conflict and stress in their later lives [73, 74]. Thus, it is possible for them to repeat these patterns of aggression and control in their intimate relationships, becoming both victims and perpetrators of IPV [72, 75]. A comprehensive understanding of how childhood trauma affects the development of violent relationships is therefore necessary for effective prevention and intervention strategies.”

**Reviewer’s comment**

10. Line 481: I would suggest making the section on more than one partner it’s own paragraph

**Authors’ response**

We appreciate your suggestion. We have reviewed this and made adjustments to ensure clarity in the revised version of the manuscript (see page 27, line 565).

**Reviewer’s comment**

11. Line 498-500: The recommendations to mitigate the impacts of IPV from this systematic review are important. I would love to see these recommendations more built out with additional citation from other literature and more detail about what types of interventions are indicated based on the findings of this specific literature review. Building this out will be very important for practitioners who want to cite this article when proposing IPV interventions.

**Authors’ response**

Thanks for your valuable suggestion. We have incorporated additional evidence supporting the recommendations in the revised version of the manuscript and our recommendations are indicated based of the reviewed literature as stated below (see page 27, lines 584-589) in the revised version of the manuscript:

“This systematic review highlights the need for interventions that mitigate IPV impacts. These interventions may include providing safe spaces for victims to seek support and counselling, as well as improving access to mental health support services [78-80]. Furthermore, evidence-based interventions, such as trauma-informed care and cognitive-behavioural therapy interventions, have shown effectiveness in reducing IPV impacts and promoting survivor well-being [80-84].”

**Reviewer’s comment**

12. Line 507-509: I would delete “potentially” from the sentence as suicide is harmful in all instances. Additionally, I would welcome inclusion of additional maladaptive coping strategies observed other than suicide.

**Authors’ response**

Your feedback is greatly appreciated. We agree and have removed the word "potentially" from the sentence in the revised version. Further, in the revised manuscript, we have included other maladaptive by adding a sentence as given below (see page 28, lines 598-599):

“Furthermore, a variety of maladaptive coping strategies may result, such as self-harm, substance abuse, and eating disorders [13, 14, 33, 89, 90].”

**Reviewer’s comment**

13. Line 517-518: I would suggest rephrasing this sentence as it implies that those who are HIV positive have “unbearable lives” which can be stereotyping for those in the HIV positive community and should be avoided.

**Authors’ response**

Thank you for your feedback. We have rephrased the sentence in the revised version as below (see page 28, lines 609-612):

“There is a significant impact on lives of IPV victims who experience sexual and reproductive health problems, including HIV infection [91, 96, 97]. These individuals often experience considerable suffering as well as many physical, emotional, and social difficulties.”

**Strength and Limitations**

**Reviewer’s comment**

14. I welcome seeing the analysis of how the search terms focused on martial/spousal relationships may have limited the study to understanding IPV against women in that population.

**Authors’ response**

Thank you for your valuable feedback about the potential limitations associated with focusing on marital/spousal relationships. We want to clarify that we made sure to include all relevant articles that examined IPV against women, irrespective of their age or marital status. Therefore, we have removed this limitation from our study. However, we want to highlight that our review primarily focuses on heterosexual women as victims of IPV. We acknowledge that this focus is a limitation of our study and we have addressed this in the revised version of the manuscript. (page 29, lines 634-636):

“Second, the focus of our systematic review is primarily on heterosexual women who are victims of IPV. Therefore, our findings may not be generalizable to individuals with diverse sexual preferences and gender identities.”

**Reviewer’s comment**

15. It would also be helpful to see here in the limitations some discussion of how the small number of articles may impact the conclusions drawn. For some of the factors or consequences that were identified they were only listed in a few of the studies included which limits the readers ability to understand these factors as inclusive.

**Authors’ response**

Thank you very much for this valuable feedback. In the revised version of the manuscript, we have addressed this limitation as below (page 30, lines 653-655):

“Another limitation of this review is the relatively small number of articles available for inclusion especially for some of the factors or consequences, which may affect the generalizability of our results.”

**Reviewer’s comment**

16. Also, can you include a limitation on the fact that the study only focused on heterosexual women victims of IPV? And does not seem to look at additional vulnerabilities with intersectional identities which seems important to consider in future research.

**Authors’ response**

Thanks for your insightful feedback and suggestion. We have included this limitation in the revised manuscript, as you below (page 29, lines 634-636)

“Second, the focus of our systematic review is primarily on heterosexual women who are victims of IPV. Therefore, our findings may not be generalizable to individuals with diverse sexual preferences and gender identities.”

**Conclusions**

**Reviewer’s comment**

17. The conclusion section could benefit from revision to strengthen the final conclusions drawn and the call to action. The recommendation for a large-scale population based study in South Asia does not to me seem linked to the findings and discussion which seemed to focus more on the need for more nuanced understandings of the factors related to IPV and how to construct interventions as a whole. The study does not seem to indicate a lack of data—rather a lack of detailed analysis of factors. I would expect to see the conclusions and recommendations for this paper more focused in this area and therefore suggest a rethinking and reframing of the conclusion.

**Authors’ response**

Thank you for your feedback and suggestion. We have reframed the conclusion section by emphasizing the need for a deeper understanding of IPV factors and the development of targeted interventions as below (pages 31, lines 659-677).

“The high prevalence of IPV was found to be significantly associated with a variety of demographic, family, community, and behavioural risk factors which emphasizes the complex nature of this public health issue. IPV has adverse effects on women, children, families, and society as a whole. Therefore, to effectively address IPV and its impact, it is necessary to develop appropriate interventions based on these risk factors and consequences. The implementation of policy driven, and appropriate interventions should be guided by a thorough understanding of IPV dynamics and tailored to the unique needs of different populations. Accordingly, this study highlights the need for further research to achieve more national level up to date data and critical analysis to explore the underlying factors and consequences of IPV, which in turn may facilitate more productive, preventative and intervention efforts in LMICs, particularly in South Asia, where IPV is particularly prevalent. Effective intervention programs and interference from social advocacy groups by targeting vulnerable group of women can also be considered to achieve SDG 5.2 by 2030.”

**Reviewer # 2**

**Reviewer’s comment**

1. The appropriate reference for the WHO definition of IPV should be the World Report on Violence and Health (Krug, Dahlberg, Mercy, Zwi, & Lozano, 2002). The authors instead cite a WHO webpage, which itself reference the World Report (pages 2-3, reference <https://apps.who.int/iris/bitstream/handle/10665/42495/9241545615_eng.pdf>

**Authors’ response**

Thank you for your feedback on the appropriate reference to the WHO IPV definition.

Following your recommendation, we have replaced the suggested reference in the revised version (see page 3, line 51- 54).

**Reviewer’s comment**

2. The methods section indicates that only 3 databases were searched. Though this meets the most basic level of standards for high-quality systematic review, it barely does so. I have some concerns that several large databases were not included and the search may have therefore missed critical literature. For example, why were standard resources such as MEDLINE/PubMed, CABI’s Global Health, Social Science Research Network (SSRN), and CABI’s Global Health not included?

**Authors’ response**

Thanks for your feedback. We understand and appreciate your concern regarding the limited number of databases searched and the potential omission of critical literature. Although we acknowledge that there are many other valuable databases in the field, such as MEDLINE/PubMed, CABI's Global Health, Social Science Research Network, and CABI's Global Health, we chose the three aforementioned databases because they are relevant and suitable for our research objectives.

In our systematic review, we utilized three databases, namely Scopus, Web of Science, and ProQuest, based on their relevance to the field of public health and the recommendations provided by subject-specific librarians. These databases are widely recognized for their coverage of a diverse range of disciplines and have been extensively used in public health research. They also include a wide range of high-quality peer-reviewed journals that meet PRISMA standards.

As we understand, the inclusion of additional databases could potentially capture more articles related to the subject. However, it is important to note that the selection of databases is a balance between comprehensiveness and feasibility, considering the time and resource constraints of the study. We believe that the combination of these three databases, along with the manual search conducted using Google Scholar, provided a robust foundation for identifying relevant studies for our systematic review.

Based on a comprehensive set of search terms and inclusion criteria across all selected databases, we conducted a thorough systematic review. The objective of our study was to identify published peer-reviewed articles that met the specific objectives and scope of the study. Despite the possibility that we may have missed some literature by not including other databases, we believe that our approach captured a significant amount of the available literature. However, we have also run a quick search in SSRN and CABI databases and it appears that all the important articles that met our inclusion criteria are already included in our current review. (Note: Scopus includes records from MEDLINE and EMBASE).

Furthermore, we followed the PRISMA guidelines strictly, which are widely regarded as the gold standard for reporting systematic reviews.

**Reviewer’s comment**

3. On page 6, line 146 the authors refer to a “manual search using Google Scholar,” but this is not described in the literature search methodology above. The fact that the Google Search did result in additional studies included suggests that only restricting the search to 3 databases could have resulted in significant missed literature.

**Authors’ response**

Thank you for your comment regarding the manual search conducted using Google Scholar. We apologize for not providing a detailed description of the manual search in the method section.

This is now included in the revised manuscript as follows (page 6, lines 130-131):

“In addition to these three databases, a manual search was conducted through Google Scholar to find more relevant studies.”

**Reviewer’s comment**

4. More detail is needed regarding how the inclusion criteria were operationalized. For example, were studies included only if they presented data from adult women ages 18 and older? What about studies that also included adolescent girls? What about partnered but unmarried women?

**Authors’ response**

Your feedback is greatly appreciated. The purpose of this study was to provide a comprehensive overview of the factors and consequences associated with IPV among women by adopting a broad approach to gather evidence from a variety of contexts and populations in the LMICs. Accordingly, we chose to consider women as a whole, without imposing any specific age limit or excluding them based on their marital status. Different age groups and marital statuses among women have been identified as contributing factors to IPV. Therefore, we were able to capture a broader range of issues by including women regardless of their age or marital status. It is possible that studies conducted on young adolescent girls or women of a specific marital status may be able to provide valuable insight into IPV within these subpopulations. However, this study focused on factors and consequences associated with IPV among women in LMICs. Future studies can investigate this issue using subgroup analysis by considering different age groups and marital status or even conducting meta-analysis on sub-groups.

**Reviewer’s comment**

5. The methods section does not describe any protocol or procedures to search the grey literature, such as searching websites for reports from multilateral and bilateral organizations and other grey literature. This omission very likely introduced bias in the systematic review approach taken by the authors.

**Authors’ response**

Thanks for providing valuable feedback regarding the omission of a protocol or procedure for searching grey literature.

As part of our predefined inclusion criteria, we limited our systematic review to published, peer-reviewed articles. This type of literature was prioritized to ensure the reliability, quality and rigor of the studies included in our review. We acknowledge that grey literature may offer additional perspectives and insights, but we have decided to exclude it from the scope of this review. The decision was driven by a variety of factors, including the need to maintain a specific focus and scope, the limited resources available, and the desire to adhere to a robust methodology.

Moreover, we would like to emphasize that the NIH Quality Assessment Tool was used in our systematic review to assess the quality of the included studies. This is a widely accepted and validated technique for evaluating the risk of bias in studies, particularly in the context of health research. By using this tool, peer-reviewed articles were able to be thoroughly evaluated for their quality and validity from a methodological perspective.

We understand that grey literature may provide additional insights and perspectives, however, we chose to focus only on peer-reviewed articles published in a peer-reviewed journal because it was aligned with our study objectives. We have added this point as a limitation point in the revied version to avoid confusion as below (page 29, lines 630-633):

“First, this review was limited to peer-reviewed journal articles published in the English language, and studies published in other languages or in non-peer-reviewed sources were not included.”

**Reviewer’s comment**

6. In the results section, the authors summarize significant associations noted in the included studies. However, no mention is made of null results. Did all of the studies find only significant associations, or did the authors only selectively report significant associations? It seems important to note null results as they indicate lack of consistent patterns of findings in the literature.

**Authors’ response**

Thank you for your valuable feedback.

In our systematic review, we were interested in identifying and summarizing significant associations related to the factors and consequences of IPV among women, since such associations provide important insights into this topic. The significance of the results was chosen because we believe they contribute directly to understanding the factors and consequences of IPV. Further, most of the studies included in this review only reported significant findings.

However, based on your feedback, we have added the following sentence in the revised version (page 22, lines 447-451).

“Most of the studies included in this current review reported only the significant factors and consequences associated with IPV. However, associations between IPV and certain factors, including ownership of a mobile phone, the type of family (joint or nuclear), sexual assertiveness, number of children, and length of marriage were not found to be significant in some of the reviewed studies [7, 25, 29, 36, 41, 47].”

**Reviewer’s comment**

7. For topics where the authors report inconsistent findings in the literature, it would be helpful if the authors included at least some detail to be able to assess what factors might account for inconsistencies. For example, the authors refer to some studies that found higher risk of IPV among women who were employed, but two studies found unemployed women had higher risk of IPV (lines 246-249). Were the studies from different geographic regions? Were the women’s ages different? Is there any information from the studies that can shed light on these patterns?

**Authors’ response**

Thanks for your insightful feedback and suggestion. Regarding the varying findings regarding the risk of IPV among employed and unemployed women, it is important to consider potential factors that may explain these discrepancies. Upon further analysis, we discovered that educational disparities might explain the inconsistent findings observed across different studies. However, it is important to note that the available literature did not provide explicit information on the influence of geographical regions or age differences on these patterns. Based on your suggestion, we have included the following explanation in the revised manuscript (page 15, lines 279-284).

“When investigating inconsistencies related to employment status and IPV, it was found that educational disparities may contribute to the inconsistent results observed across different studies. Most of the women who were included in the studies that revealed that employed women were at increased risk of IPV had a primary education or no education. Conversely, most of the women who participated in studies showing that working women are less likely to develop IPV had tertiary or post-secondary education.”

**Reviewer’s comment**

8. Similarly, I urge the authors to ensure the comparison or reference group is clear in their summary of findings. For example, the statements about evidence related to marital status (lines 266-268): is being separated or divorced and in a polygamous marriage associated with higher risk of IPV compared to currently-married women? Never-married women? Some other comparison?

**Authors’ response**

Thank you for this feedback. The statement regarding evidence related to marital status has been re-written to improve clarity as below (page 16, lines 303-306):

“In two studies, it has been shown that women who have been separated or divorced [44] or involved in a polygamous marriage are at a higher risk of IPV [29] as compared to those who are currently married, widowed, or in a monogamous marriage.”

**Reviewer’s comment**

9. I disagree with the author’s characterization of literature that assesses women’s individual attitudes about IPV as reflecting “community level risk factors” (lines 291-301). Studies that measure a woman’s attitudes or beliefs about violence reflect individual risk factors, not community risk factors. Those individual attitudes may be influenced by community norms and values. However, if the studies did not measure community norms and values and instead measured individual attitudes, that reflect individual risk factors nonetheless.

**Authors’ response**

Your feedback is highly appreciated. We agree with you that studies that assess a woman's attitudes and beliefs regarding violence primarily reflect individual factors rather than community-level factors. Following this feedback, we have revised the relevant section as below (see page 16, lines 307-316):

“Attitudes toward IPV justification: Community attitudes and norms around the acceptability of violence against women were identified as risk factors for IPV in several studies. A study by Sabri, Renner [44] showed that Indian women who accept violence have a higher chance of suffering physical IPV and injuries caused by IPV. In a study of Indonesian women, Hayati, Högberg [47] found that women who agreed with the “good wife obey her husband” and “a man should show who is boss” were at a higher risk of experiencing sexual violence in relation to the women who disagreed. Further, this study determined that women who justified male violence were more likely to experience both physical and sexual violence. IPV was higher among Kenyan women when they believed their partners were justified in beating them than those who felt their partners were not justified in beating them [23].”

**Reviewer’s comment**

10. I also object to the author’s characterization of social support as a “protective factor” by virtue of the fact that its absence was found to be associated with higher risk of IPV in one study (lines 302-303). The two are not necessarily interchangeable. A more accurate assessment would be to indicate that lack of social support is a risk factor for IPV.

**Authors’ response**

Thank you for your feedback regarding the characterization of social support as a "protective factor" in relation to IPV. We acknowledge that it is important to clarify the distinction between social support as a protective factor and its absence as a risk factor for IPV. In the revised manuscript, we have modified the sentence accordingly to incorporate you feedback as follow (See page 18, lines 351-353):

“Lack of social support was identified as a risk factor of IPV in a study conducted in Israel, indicating that women with limited social support had greater exposure to IPV [50].”

**Reviewer’s comment**

11. Similarly, the description of the findings from the Sabri, Renner study (lines 304-305) bear some consideration. Did the study find that women who had more severe physical IPV were more likely to seek formal or informal help? If so, that does not suggest that help-seeking is a risk factor for IPV. Some elaboration of the findings of this particular study are warranted**.**

**Authors’ response**

Thank you for your comments and valuable suggestion. According to this study, women who sought formal or informal help were more likely to experience severe IPV than women who did not seek any help. Consequently, this highlights the complex relationship between help-seeking behaviours and the severity of IPV experienced by women. To ensure a clear interpretation of the findings of this study, this sentence has been revised and moved under behavioural risk factors in the revised version as below (See page 19, lines 376-378):

“Help seeking behaviour: Sabri, Renner [44] concluded that Indian women who sought formal or informal help were more likely to experience severe physical IPV than women who did not seek any help. Therefore, help-seeking behaviour may be associated with higher levels of IPV.”

**Reviewer’s comment**

12. The summary of the literature for “behavioural risk factors” appears to confuse risk factors with consequences. Several of the studies summarized appear to refer to what might have been mental health impacts of IPV rather than risk factors. This should be clarified.

**Authors’ response**

Thank you for this valuable comment. We truly understand the need for further clarification regarding the distinction between behavioural risk factors and consequences in the summary of literature related to IPV. However, we would like to clarify that behavioural risk factors, including alcohol usage, psychological well-being and controlling behaviour are indeed risk factors for IPV rather than consequences of IPV.

Firstly, extensive research has consistently shown an association between alcohol consumption and an increased risk of IPV perpetration and victimization. The use of alcohol can impair judgment, reduce inhibitions, and increase aggression, which may contribute to violent behaviours within intimate relationships. In our review paper, we have included alcohol usage as a behavioural risk factor because we believe it directly affects IPV occurrence.

Secondly, psychological well-being, specifically depression symptoms, is considered to be a risk factor for IPV. Although IPV is associated with adverse mental health consequences, including depression, it is important to note that depressive symptoms can also contribute to IPV. Depressive symptoms can make it difficult for individuals to effectively manage their emotions, communicate their needs, and establish healthy boundaries, all of which can increase the risk of experiencing IPV in intimate relationships. Thus, including psychological well-being, such as depression symptoms, as a risk factor in our review paper is justified since it explores the connection between pre-existing mental health issues and IPV.

Lastly, controlling behaviour has been recognized as a significant behavioural risk factor for IPV. IPV can be perpetrated and increased significantly through control-oriented behaviours such as jealousy and unfaithfulness. Considering that these behaviours are associated with an imbalance of power and control within the relationship, they are considered risk factors for IPV occurrence. Therefore, the inclusion of controlling behaviour as a behavioural risk factor is consistent with the fact that it plays a critical role in the prediction of IPV (McClintock et al., 2021).

(Ref: McClintock HF, Trego ML, Wang EM. Controlling Behavior and Lifetime Physical, Sexual, and Emotional Violence in sub-Saharan Africa. Journal of Interpersonal Violence. 2021;36(15-16):7776-801)

**Reviewer’s comment**

13. I am also inclined to object to the use of the term “behavioural risk factors” to refer to phenomena like depression and other mental health issues. These are not always characterized behaviourally.

**Authors’ response**

Thank you for your comment. We understand your concern regarding the use of the term "behavioural risk factors" to describe issues such as depression. We would like to provide clarification on the inclusion of psychological well-being, including depression symptoms, as a behavioural risk factor for IPV (Capaldi et al., 2012).

(Ref: Capaldi, D. M., Knoble, N. B., Shortt, J. W., & Kim, H. K. (2012). A Systematic Review of Risk Factors for Intimate Partner Violence. *Partner Abuse*, *3*(2), 231-280. <https://doi.org/10.1891/1946-6560.3.2.231> )

Although depression and mental health problems are not exclusively characterized by observable behaviours, it is important to acknowledge that they may appear behaviourally and influence IPV. We use the term "behavioural risk factors" in our review to refer on factors that have a behavioural component or can be expressed as behaviours that have the potential to increase risk of IPV.

For instance, depression symptoms can affect an individual's behaviour, interpersonal interactions, and emotional regulation, thereby affecting their intimate relationships. The presence of depression may result in low self-esteem, difficulties in communicating and solving problems, as well as an increased risk of experiencing abusive behaviours (Dutton & Karakanta, 2013; Petrović, 2017; Sharma et al., 2019). Thus, the inclusion of psychological well-being, including depression symptoms, as a behavioural risk factor is based upon the understanding that these internal states may lead to behavioural problems and contribute to IPV risk.

(Ref: Dutton, D. G., & Karakanta, C. (2013). Depression as a risk marker for aggression: A critical review. *Aggression and Violent Behavior*, *18*, 310-319. <https://doi.org/10.1016/j.avb.2012.12.002>; Petrović, M. (2017). The impact of depression on the human personality. *European Psychiatry*, *41*(S1), S538-S538. <https://doi.org/10.1016/j.eurpsy.2017.01.740>; Sharma, K. K., Vatsa, M., Kalaivani, M., & Bhardwaj, D. N. (2019). Mental health effects of domestic violence against women in Delhi: A community-based study. *Journal of Family Medicine and Primary Care*, *8*, 2522 - 2527. <https://www.ncbi.nlm.nih.gov/pmc/articles/PMC6691463/pdf/JFMPC-8-2522.pdf>)

**Reviewer’s comment**

14. The authors assess study quality of the included studies and provide that information in a brief table, but study quality information does not appear to factor into the summary or conclusions. How was study quality taken into account in synthesizing research findings? Did it inform the authors in assessing consistencies or inconsistencies in the literature?

**Authors’ response**

We appreciate your valuable feedback about the study quality assessment. In our systematic review, we used the NIH Quality Assessment Tool to evaluate the quality of the included studies, as stated in the method section. We assessed various aspects, including population specification, participation rate, sample size justification, and assessor blinding status, among others.

Even though it may appear that study quality information has no direct influence on the summary or conclusions, it played a substantial role in our synthesis and interpretation of the research findings. The quality assessment was primarily conducted to ensure all studies were of adequate quality for inclusion. Although no studies were excluded on this basis, careful consideration was given to the two studies that did receive ‘poor’ quality to ensure that the factors contributing to the poor quality were considered during the integration of the results. A separate table was created to provide transparency and facilitate an understanding of the methodological rigor of each study.

Furthermore, in the Results section, we provided a summary of the study quality assessment findings, showing that twenty-one studies were rated as good, nine studies as fair, and two studies as poor. Furthermore, we discussed the specific reasons for the poor ratings of the two studies conducted by Pahn and Yang [22] and Memiah, Ah Mu [23]. Considering their adequate power and use of cross-sectional self-reported opinion surveys, we decided to include these studies in the review. Further assessment of the quality of the findings of the reviewed studies and consistency including bias and heterogeneity can be done through a meta-analysis which was not the objective of the current study. This could be considered in future studies.

**Reviewer’s comment**

15. More detail could be provided regarding the gaps in the research – what are the areas that need further investigation? There is a very brief paragraph at the end that provides a somewhat perfunctory set of recommendations for future research. More could be said that is directly informed by the findings of the systematic review.

**Authors’ response**

Thanks for this feedback. On the basis of you suggestion, we have re-written this paragraph in the revised manuscript to provide a more comprehensive overview of the areas that require further research as below (pages 32, lines 692-713):

“The IPV prevalence rate was found to be relatively high in South Asian LMICs. However, this review highlights a significant gap in the existing literature, as only a few studies have been undertaken in this region despite the high prevalence of IPV. From this review, it is clear that, to date, only a few studies have been conducted to explore women’s attitudes toward IPV in LMICs. Thus, there is a need to conduct rigorous large-scale population-based studies that explore women’s attitude toward IPV in LMICs along with the risk factors and consequences of such violence on women’s mental and physical health, the marital relationship, children, families, communities, and wider society. Based on this study findings, it can be concluded that education plays a significant role in reducing IPV. Nevertheless, it is important to note that education plays a mediation role as it often indirectly related to other contributing factors of IPV, such as gender inequality, women’s empowerment, and cultural norms. Therefore, further research is needed to explore the complex interrelation between education and these factors and how they collectively influence the prevalence and consequences of IPV in LMICs. This includes exploring the path analysis by which level of education influence IPV, and how they interact with other socio-demographic determinants. Additionally, future research should focus on developing and evaluating educational interventions and empowerment programs designed to reach vulnerable individuals, families, and communities. The aim of the interventions should be to promote empowerment women through education and gender equality and enhance mental health and well-being. There is also a need to conduct a large scale qualitative and mixed method studies linking appropriate theoretical model to understand the perceptions, subjective feelings, and experiences of IPV among women in LMICs, especially among Asian women.”

**Reviewer’s comment**

16. I was disappointed to find that the manuscript does not include a summary table with the overall findings related to risk factors and consequences. Table 1 provides an overview of the studies, but it does not include summary findings, which seems to be the most important information.

**Authors’ response**

Thank you for the feedback on the expectation for a summary table that summarizes the overall findings related to risk factors and consequences in our manuscript. We would like to clarify that our manuscript includes a summary table that summarizes our overall findings of risk factors, consequences, and implications in Table 2 entitled “Critical Findings and Implications” on page 33 & 34. This table provides a comprehensive summary and facilitates understanding of the main results presented in our systematic review.

**Reviewer’s comment**

17. There are a number of grammatical issues throughout the manuscript that make some sentences difficult to follow (especially due to several sentence fragments and some run-on sentences). I recommend working with a strong language editor to recommend revisions to the paper.

**Authors’ response**

Thanks for your feedback. The updated manuscript has undergone a comprehensive review by language editors to address any English language and grammatical concerns. Additionally, all authors have reviewed the revised version to rectify language and grammatical issues.
