# Supplementary material for: Factors and consequences associated with intimate partner violence against women in low- and middle-income countries: A systematic review
Source: PLoS One. 2023 Nov 8;18(11):e0293295. doi: 10.1371/journal.pone.0293295 (PMC10631698; doi:10.1371/journal.pone.0293295)
Supplement: Attachment — Submitted filename: Response to Reviewers.docx [file pone.0293295.s004.docx]

19^th^ September, 2023

Abraham Salinas-Miranda
Academic Editor,
PLOS ONE

RE: PLOS ONE – Decision on Manuscript ID PONE-D-23-08947

Dear Editor,

We appreciate the opportunity to revise our manuscript titled "Factors and Consequences associated with Intimate Partner Violence against Women in Low- and Middle-Income Countries: A Systematic Review" for publication in PLOS ONE.

We have thoroughly attended to the concerns raised by the academic editor and reviewers and have integrated the required modifications into the original manuscript. Our comprehensive revision process is detailed in the point-by-point response provided below. In the revised version, we have highlighted the reviewers' comments in blue and our responses in black font for clarity.

Furthermore, we confirm that all authors have thoroughly reviewed the revised version of the manuscript. We have made some changes to the revised version in order to improve its clarity.

We sincerely thank you for considering our paper and appreciate the valuable suggestions and feedback provided by the academic editor and reviewers. Their comments and suggestions have significantly improved the manuscript quality. We hope that the revised version will meet the standards for publication in your esteemed journal.

Thank you for your kind consideration.

Kind regards,

Lakma Gunarathne

Swinburne University of Technology

Victoria, 3122, Australia.

**Co-authors:**

1. Jahar Bhowmik, Department of Health Science and Biostatistics, Swinburne University of Technology, Australia.

2. Pragalathan Apputhurai, Department of Health Science and Biostatistics, Swinburne University of Technology, Australia.

3. Maja Nedeljkovic, Department of Psychological Sciences, Swinburne University of Technology, Australia.

**Factors and Consequences associated with Intimate Partner Violence against Women in Low- and Middle-Income Countries: A Systematic Review**

(PONE-D-23-08947)

(Please Note: Page numbers and line numbers in this document are based on Revised Manuscript with Track Changes file)

**Reviewer # 1**

**Abstract**

1. Line 28: Please clarify the statement “relatively high prevalence rates” as it is not clear what you are comparing this to? Relative to what? I would suggest choosing more precise language

**Authors’ response**

Thank you for your feedback about the clarification of the statement regarding "relatively high prevalence rates". In response to your suggestion, we have revised this section. This sentence has been updated as, " Intimate Partner Violence (IPV) is a global public health issue, with notably high prevalence rates observed within Low-and Middle-Income Countries (LMICs)."

(see page 2; lines28-29 in the revised manuscript with track changes)

2. Line 29: should it read “within” LMIC’s rather than “among”?

**Authors’ response**

Thanks for your suggestion. We have revised the sentence in the revised version as follows:

“Intimate Partner Violence (IPV) is a global public health issue, with notably high prevalence rates observed within Low-and Middle-Income Countries (LMICs).”

(see page 2; line 29 in the revised manuscript with track changes)

3. Line 39: You state “The findings indicate alarmingly high prevalence of IPV against women in many LMICs” This is confusing because it is not clear what this is being compared to? i.e. it is alarmingly high compared to what? Additionally quantifying the prevalence of IPV is not an explicit aim of this systematic review and therefore this sentence does not make sense to include in the abstract and is misleading to the study findings. Suggest rephrasing.

**Authors’ response**

We appreciate your insightful feedback. We have reviewed this, and have rephrased the sentence as follow:

“In this study, several risk factors were identified including lower levels of education, marriage at a young age, poor wealth indices, rural residential areas, and acceptance of gender norms that contribute to the prevalence of IPV in LMICs. It is essential to address these factors through effective preventive policies and programs.”

(See page 2; lines 39-43 in the revised manuscript with track changes)

**Introduction**

**Reviewer’s comment**

4. Overall the Introduction is still quite repetitive and not well organized. The topics covered in the introduction jump around significantly and it would overall benefit from additional editing.

**Authors’ response**

Thank you for your valuable feedback and suggestion.

Based on your suggestion, we have edited the introduction section and have removed the repetitions to make it concise in the revised version of the manuscript

(see pages 3-4, lines 54, 57-64, 66, 76-77, 86, 87-88, 94-95, 99, 100-101 in the revised manuscript with track changes).

**Reviewer’s comment**

5. Line 50: “IPV is known as” should read “defined as” as you are sharing a definition

**Authors’ response**

Thank you for your attention and picking up this discrepancy. Following your suggestion, we have replaced the word “known as” by “defined as” in the revised version.

(See page 3; line 54 in the revised manuscript with track changes).

**Reviewer’s comment**

6. Line 61-62: “Women are at a higher risk of experiencing IPV compared to men” To make this statement the rate of IPV of risk for men needs to be cited to, as of now it’s not clear what you’re comparing to

**Authors’ response**

Thank you for your insightful comment and suggestion. We have incorporated your suggestions and cited the rate of IPV of risk for men in the revised version as below:

“Therefore, women are at a higher risk of experiencing IPV compared to men, with nearly 1 in 7 men experiencing IPV [5, 6]”.

(see page 3; line 66 in the revised manuscript with track changes)

***References:***

*5.	World Health O. Global and regional estimates of violence against women: prevalence and health effects of intimate partner violence and non-partner sexual violence. Geneva: World Health Organization; 2013 2013.*

*6.	Jenabi E, Khazaei S. Prevalence rate of intimate partner violence by WHO region: an appraisal by current data. Asian Journal of Health Sciences. 2018;4(1).*

**Reviewer’s comment**

7. Line 93-96: This sentence is very confusing. Rephrase

**Authors’ response**

Thank you for the valuable input in enhancing the clarity of our manuscript. We have rephrased the sentence as follow.

“To the authors’ knowledge, no effort has been made to aggregate and systematically review both factors associated with IPV and consequences of IPV against women in LMICs. This study aims to provide a more comprehensive understanding of this prevalent public health concern.”.

(see page 4; lines 98-101 in the revised manuscript with track changes)

**Materials and Methods**

**Reviewer’s comment**

8. Line 109: It would be helpful to include more information about why these data bases were selected and why only 3 were used.

**Authors’ response**

Thanks for your feedback. In response, we have added the following sentences.

“These databases are widely recognized for their coverage of a diverse range of disciplines and have been extensively used in public health research. They also include a wide range of high-quality peer-reviewed journals that meet PRISMA standards.”

(see page 5; lines 115-118 in the revised manuscript with track changes)

**Reviewer’s comment**

9. Line 158: Please state explicitly that no studies were removed following the quality assessment**.**

**Authors’ response**

Thank you for suggesting. We have incorporated the following sentence in the revised version.

“No studies were removed following quality assessment.”

(See page 7; lines 167-168 in the revised manuscript with track changes)

**Results**

**Reviewer’s comment**

10. Line 242: should read “experiencing”

**Authors’ response**

Thanks for your suggestion. We have revised the sentence as follows in the revised version

“Studies also showed that women’s employment status is significantly associated with IPV against women in LMICs, with the risk of **experiencing** IPV higher for the women who were currently employed compared to those who were unemployed.”

(see page 13; line 247 in the revised manuscript with track changes)

**Reviewer’s comment**

11. Line 382: Is the reference to women from the country of South Africa or the southern part of the African continent? Not sure if south not being capitalized is a typo or not?

**Authors’ response**

Thanks for pointing out this typo. To indicate women from South Africa, it should be capitalized. This has been corrected in the revised manuscript as follow:

“One study found that **South African** women who had experienced maternal emotional IPV had lower cognitive, language, or motor composite scores for their children at age two.”

(see page 20; line 393 in the revised manuscript with track changes)

**Discussion**

**Reviewer’s comment**

12. Overall, the Discussion could benefit from revision with a particular focus on how risk factors may interact throughout the analysis. For example, I note on Line 408-410 that in the list of reasons why lower education may lead to IPV the other risk factors related to environment and access to education in the first place are not integrated into the discussion and analysis. I believe that there are many interactions between both the risk factors and consequences identified in the analysis and the paper would be greatly strengthened by including more nuanced discussion of these interactions.

**Authors’ response**

Thank you for your feedback. We have incorporated your suggestions and included more nuanced discussion in the revised version as follow:

“Further, in many LMICs, factors such as prevailing gender norms within rural communities as well as limited access to quality education facilities can further contribute to a lower educational level among women, increasing their vulnerability to IPV [31, 53, 54].”

(See page 21; lines 422-424 in the revised manuscript with track changes)

***References***

*31.	Sanni Y, Hudani A, Buh A, Ghose B. Prevalence and Predictors of Intimate Partner Violence Among Married Women in Egypt. Journal of Interpersonal Violence. 2021;36(21-22):10686-704.*

*53.	Nabaggala MS, Reddy T, Manda S. Effects  of rural–urban residence and education on intimate partner violence among women in Sub-Saharan Africa: a meta-analysis of health survey data. BMC Womens Health. 2021;21(1):149*

*54.	Oluwagbemiga A, Johnson A, Olaniyi M. Education and Intimate Partner Violence Among Married Women in Nigeria: A Multilevel Analysis of Individual and Community-Level Factors. J Interpers Violence. 2023;38(3-4):3831-63.*

Also, based on your suggestion, we have revised the discussion section incorporate more nuanced discussion on the interaction between risk factors and consequences of IPV.

(See pages 20-27; lines 413-414, 416, 422-424, 460-468, 489-492, 500-501, 504, 522-523, 525-533,556-567in the revised manuscript with track changes)

**Reviewer’s comment**

13. Line 438-447: In this paragraph it may be helpful to also consider why older women, if they experience IPV may not say anything or report it? What role might stigma or social norms play in less reporting of IPV by older women?

**Authors’ response**

Thank you for your feedback and suggestion. According to your suggestion, we have added the following text in the revised version of the manuscript

“However, it is possible that older women report less IPV because of additional barriers, such as the stigma associated with IPV and societal expectations for long-term marriages [64]. Social norms that discourage openly discussing marital conflicts or seeking outside assistance may also discourage older women from reporting IPV incidents [64, 65]. Consequently, IPV may be at a lower prevalence among older women as compared to younger women. Thus, interventions aimed at reducing IPV should be targeted at women in younger age groups, particularly those who are at risk of or have experienced child marriage. Additionally, older women also face different challenges, so interventions should also emphasize creating a safe environment for them to seek support and report IPV incidents.”

(see page 22-23; lines 460-468 in the revised manuscript with track changes)

***References***

*64.	Meyer SR, Lasater ME, García-Moreno C. Violence against older women: A systematic review of qualitative literature. PLoS One. 2020;15(9):e0239560.*

*65.	Band-Winterstein T. Aging in the Shadow of Violence: A Phenomenological Conceptual Framework for Understanding Elderly Women Who Experienced Lifelong IPV. Journal of Elder Abuse & Neglect. 2015;27(4-5):303-27.*

**Reviewer’s comment**

14. Line 463-473: In discussion of the area where women live I don’t see discussion of economics or educational level, as they may be tied to living in a more rural location discussed though I would imagine that that could also have an impact on the risk of IPV in rural areas?

**Authors’ response**

We appreciate your suggestion. We have included the following statement in the revised version.

“Several factors may contribute to this, including low socioeconomic status and limited educational opportunities, which are common among the women reside in rural areas [53, 68]. Additionally, in rural areas women lack access to social support and media, and they tend to accept social and religious norms that emphasise male dominance [8, 10, 31].”

(see page 23-24; lines 489-492 in the revised manuscript with track changes)

***References***

*8.	Bhowmik J, Biswas RK. Married Women's Attitude toward Intimate Partner Violence Is Influenced by Exposure to Media: A Population-Based Spatial Study in Bangladesh. Int J Environ Res Public Health. 2022;19(6).*

*10.	Coll CVN, Ewerling F, García-Moreno C, Hellwig F, Barros AJD. Intimate partner violence in 46 low-income and middle-income countries: an appraisal of the most vulnerable groups of women using national health surveys. BMJ Glob Health. 2020;5(1):e002208.*

*31.	Sanni Y, Hudani A, Buh A, Ghose B. Prevalence and Predictors of Intimate Partner Violence Among Married Women in Egypt. Journal of Interpersonal Violence. 2021;36(21-22):10686-704.*

*53.	Nabaggala MS, Reddy T, Manda S. Effects  of rural–urban residence and education on intimate partner violence among women in Sub-Saharan Africa: a meta-analysis of health survey data. BMC Womens Health. 2021;21(1):149.*

*68.	Speizer IS. Intimate partner violence attitudes and experience among women and men in Uganda. J Interpers Violence. 2010;25(7):1224-41.*

**Reviewer’s comment**

15. Line 499: Was the research focused on women who have partners outside of marriage or men with multiple partners? The phrasing of this sentence is confusing to me

**Authors’ response**

Thank you for bringing this to our attention. The following statement was added in the revised version to improve clarity.

“Furthermore, IPV is more likely to occur when women have more than one sexual partner in addition to their husband.”

(see page 25; lines 522-523 in the revised manuscript with track changes)

**Reviewer’s comment**

16. Line 502 and 507: “husbands with adverse characteristics” is a weird phrasing to me. I would suggest rephrasing

**Authors’ response**

Your feedback is greatly appreciated. We have revised the statement in the revised version as follow:

“The husband's undesirable traits and troublesome conduct, such as excessive drinking, as well as negative control behaviours (jealousy, suspicion, unfaithfulness, etc.), can also contribute to impulsive reactions to verbal arguments or conflicts between husband and wife, leading to IPV [30, 38, 71, 80]. The findings indicate that social and community-based interventions are necessary by targeting the vulnerable group of women who witnessed violence during childhood, experienced extra marital relationships, or whose husbands exhibited troublesome behaviours.”

(see page 25; lines 525-533 in the revised manuscript with track changes)

**Reviewer # 3**

**Reviewer’s comment**

1. From the previous revision: "Reviewer’s comment not addressed: 12. The summary of the literature for “behavioural risk factors” appears to confuse risk factors with consequences. Several of the studies summarized appear to refer to what might have been mental health impacts of IPV rather than risk factors. This should be clarified."

   Considering the previous review comment, the Conclusion section on "Behavioural risk factors" (lines 356-378) needs to be revised. The only behaviors that appear to have some evidence for risk factors for experiencing IPV appear to be "partner's controlling behaviors" and "women help-seeking behaviors". Behaviors such as "women's alcohol use" and "psychological well-being" (not a behavior, but a mental health state) are not supported by study findings as risk factors.

   The authors wrote a detailed explanation for why risk factors such as psychological well-being factors were not outcomes but risks. Nevertheless, the articles cited examined the directionality of IPV as risk to mental health outcomes (e.g., depression). For instance, in "Behavioural risk factors > Psychological well-being" (Lines 367-369), the paper cites reference 27 and 36. The data from these studies do not support psychological well-being as risk factor but as outcome as follows:

   Ref. 27: Jiwatram-Negrón, T., Michalopoulos, L. M., & El-Bassel, N. (2018). The syndemic effect of injection drug use, intimate partner violence, and HIV on mental health among drug-involved women in Kazakhstan. Global social welfare : research, policy & practice, 5(2), 71–81. <https://doi.org/10.1007/s40609-018-0112-1>. This reference (reference number 27) found that the syndemic of IPV, substance abuse, and HIV increased 15.5 fold odds of reporting depression. Not the other way around. IPV was not significantly correlated with injected drugs (Jiwatram-Negron's Table 2: r=-0.047 between IPV and injected drugs). On Table 3 of the same article, victim's binge drinking was not significantly associated with depression and suicide either.

   In the subsection: "Behavioural risk factors > Alcohol usage" (Lines 360-361), the paper cites reference 27 as follows: "Alcohol usage: Hazardous drinking among women was found to be significantly associated with physical and/or sexual IPV experienced by south African women [27]." This sentence must be deleted as it is not factual.

   The data from ref 27 do not support women's substance abuse as risk factor for IPV. Jiwatram-Negron only reported correlation, which was not significant. Correlation should not be confused with risk factor (i.e., the word risk factor implies a directionality). When Jiwatram and colleagues discussed women's substance abuse and IPV, they used "injected drugs" not "hazardous drinking" like it was mentioned in the paper here. Jiwatram-Negron and colleagues used the lens of syndemic theory which states that these conditions act synergistically (correlated, bidirectional, interaction effects). They are part of a syndemic of IPV+substance abuse+HIV. The authors need to clarify that so they do not give the impression that women's alcohol abuse disorders increased the risk for experiencing IPV. Instead, women's substance use disorders co-occur with IPV. IPV victims use substances to numb the effect of traumatic experiences (i.e., IPV) and their perpetrators use their drinking as a way to exert further control over the victims. Alcohol use disorder may increase the vulnerability to become a victim of abuse due to their impaired decision-making. However, the study by Jiwatram and colleagues did not test that as they used "injected drugs" (correlation not significant any way).

   In the section "Behavioral risk factors > Psychological well-being" (line 367-369), the following sentence was written: "Psychological well-being: IPV was found to be negatively 367 associated with psychological well being [36];" This sentence must be deleted as it is not supported by the study data. In Ref. 36 (McClintock, H. F., Trego, M. L., & Wang, E. M. (2021). Controlling Behavior and Lifetime Physical, Sexual, and Emotional Violence in sub-Saharan Africa. Journal of interpersonal violence, 36(15-16), 7776–7801), McClintock and colleagues did not examine psychological well-being as risk factor. Instead, they used DHS data (cross-sectional) to assess partner controlling behaviors and their associations with IPV.

   Both studies (ref 27 and 36) were cross-sectional. Causality cannot be inferred. This is a major limitation of many studies reviewed.

   Perhaps, the reference was 35 and not 36. However, reference 35 was not assessing psychological well-being either as risk but as an outcome instead. John NA, Kapungu C, Sebany M, Tadesse S. Do Gender-Based Pathways Influence Mental Health? Examining the Linkages Between Early Child Marriage, Intimate Partner Violence, and 736 Psychological Well-being among Young Ethiopian Women (18–24 years Old). Youth and Society.
   737 2022.

**Authors’ response**

We are grateful to the reviewer for their thorough review and invaluable feedback. Based on your insightful suggestions, we have diligently revised our manuscript to improve both its accuracy and clarity.

In regard to the sections on psychological well-being and alcohol use under the category of behavioral risk factors, we originally cited John, 2022 (John NA, Kapungu C, Sebany M, Tadesse S. Do Gender-Based Pathways Influence Mental Health? Examining the Linkages Between Early Child Marriage, Intimate Partner Violence, and 736 Psychological Well-being among Young Ethiopian Women (18–24 years Old). Youth and Society.737 2022) and Jina, 2012 (Jina R, Jewkes R, Hoffman S, Dunkle KL, Nduna M, Shai NJ. Adverse Mental Health Outcomes Associated With Emotional Abuse in Young Rural South African Women: A Cross-Sectional Study. Journal of Interpersonal Violence. 2012;27(5):862.), respectively. An earlier version of the manuscript without track changes contains the correct references.

Additionally, we want to clarify that the study by Jiwatram,2018 (Jiwatram-Negrón, T., Michalopoulos, L. M., & El-Bassel, N. (2018). The syndemic effect of injection drug use, intimate partner violence, and HIV on mental health among drug-involved women in Kazakhstan. Global social welfare : research, policy & practice, 5(2), 71–81. <https://doi.org/10.1007/s40609-018-0112-1>), which was mentioned by the reviewer, has been correctly cited under the consequences of IPV as follow:

“One study also reported that depression was higher among drug-involved IPV suffering women than in drug-involved women who did not experience IPV in Kazakhstan [28]” (see page 18; line 348-349)

***Reference***

*Jiwatram-Negrón, T., Michalopoulos, L. M., & El-Bassel, N. (2018). The syndemic effect of injection drug use, intimate partner violence, and HIV on mental health among drug-involved women in Kazakhstan. Global social welfare : research, policy & practice, 5(2), 71–81.* [*https://doi.org/10.1007/s40609-018-0112-1*](https://doi.org/10.1007/s40609-018-0112-1)*)*

Based on the feedback and suggestion provided by the reviewer, we have decided to retain only the husband's alcohol use, controlling behavior, and help-seeking as behavioral risk factors, and revised this section in the revised version as follow:

“*Husband’s Alcohol usage:* Sabri, Renner [44] found that Indian women’s alcohol usage was not significantly associated with IPV. However, the study revealed that their husbands’ alcohol usage was related to developing physical violence and severe IPV related injuries. In addition, four studies also found that a partner’s adverse alcohol consumption resulted in a higher prevalence of IPV against women in LMICs [30, 38, 41, 47].

*Controlling behaviour:* Controlling behaviour was found to be associated with IPV in several studies, indicating that women with negative controlling behaviours such as jealousy and unfaithfulness were more likely to experience any form of IPV [37]. In addition, two studies have shown that husbands’ or partners’ adverse characteristics including jealousy, suspicion, unfaithfulness, fighting with other men and emotionally and sexually abusive behaviours had also increased the likelihood of women’s exposure to IPV [44, 47].

*Help seeking behaviour:* Sabri, Renner [44] concluded that Indian women who sought formal or informal help were more likely to experience severe physical IPV than women who did not seek any help.

(see page 16-17; lines 311-333 in the revised manuscript with track changes)

***References***

*30. Mootz JJ, Basaraba CN, Corbeil T, Johnson K, Kubanga KP, Wainberg ML, et al. Armed conflict, HIV, and syndemic risk markers of mental distress, alcohol misuse, and intimate partner violence among couples in Uganda. Journal of Traumatic Stress. 2021;34(5):1016-26.*

*37. McClintock HF, Trego ML, Wang EM. Controlling Behavior and Lifetime Physical, Sexual, and Emotional Violence in sub-Saharan Africa. Journal of Interpersonal Violence. 2021;36(15-16):7776-801.*

*38. Oluwole EO, Onwumelu NC, Okafor IP. Prevalence and determinants of intimate partner violence among adult women in an urban community in lagos, southwest nigeria. Pan African Medical Journal. 2020;36:1-16.*

*41. Ibrahim Z, Ahmed WS, El-Hamid S, Hagras A. Intimate partner violence among Egyptian pregnant women: incidence, risk factors, and adverse maternal and fetal outcomes. Clinical and experimental obstetrics & gynecology. 2015;42(2):212-9.*

*44. Sabri B, Renner LM, Stockman JK, Mittal M, Decker MR. Risk Factors for Severe Intimate Partner Violence and Violence-Related Injuries Among Women in India. Women & Health. 2014;54(4):281-300.*

*47. Hayati EN, Högberg U, Hakimi M, Ellsberg MC, Emmelin M. Behind the silence of harmony: risk factors for physical and sexual violence among women in rural Indonesia. BMC Womens Health. 2011;11(1):52.*

Furthermore, we have introduced new mental health consequences of IPV, which specifically address hazardous drinking behavior and drug use.

*“Hazardous drinking behaviour and drug use:* Jina, Jewkes [27], reported that South African women who experienced physical and/or sexual IPV with emotional abuse were much more likely to engage in hazardous drinking behaviour and use illicit drugs*.*”

(see page 19; lines 377-379 in the revised manuscript with track changes)

***References***

*27. Jina R, Jewkes R, Hoffman S, Dunkle KL, Nduna M, Shai NJ. Adverse Mental Health Outcomes Associated With Emotional Abuse in Young Rural South African Women: A Cross-Sectional Study. Journal of Interpersonal Violence. 2012;27(5):862.*

**Reviewer’s comment**

2. This reviewer also agrees with previous review that the section keeps mixing behavioral factors (conduct or life style or addictions) with mental health factors. Behavioral health has more to do with the specific actions people take. It's about how people respond in different life scenarios. Two people who are experiencing depression may react in different ways. Mental health, on the other hand, has more to do with thoughts and feelings (e.g., anxious trait, anxious state; depression; PTSD). Mental health issues can have behavioral manifestations, but those must be delineated in a review like this.

**Authors’ response**

Thank you very much for your thoughtful feedback.

As a result of the reviewer's comments, we agree that a clear distinction must be made between behavioral factors and mental health factors in our manuscript.

This concern has been addressed in the revised version by making the necessary revisions. We have moved the previously confusing behavioral risk factors, such as psychological well-being and hazardous alcohol consumption behaviour, to the section describing the consequences of IPV in the revised version as follow:

*“Hazardous drinking behaviour and drug use:* Jina, Jewkes [27], reported that South African women who experienced physical and/or sexual IPV with emotional abuse were much more likely to engage in hazardous drinking behaviour and use illicit drugs.”

(see page 19; lines 377-379 in the revised manuscript with track changes)

***References***

*27. Jina R, Jewkes R, Hoffman S, Dunkle KL, Nduna M, Shai NJ. Adverse Mental Health Outcomes Associated With Emotional Abuse in Young Rural South African Women: A Cross-Sectional Study. Journal of Interpersonal Violence. 2012;27(5):862.*
